# Potential Food and Nutraceutical Applications of Alginate: A Review

**DOI:** 10.3390/md20090564

**Published:** 2022-09-04

**Authors:** Decheng Bi, Xu Yang, Lijun Yao, Zhangli Hu, Hui Li, Xu Xu, Jun Lu

**Affiliations:** 1 Shenzhen Key Laboratory of Marine Bioresources and Ecology, College of Life Sciences and Oceanography, Shenzhen University, Shenzhen 518060, China; 2 College of Physics and Optoelectronic Engineering, Shenzhen University, Shenzhen 518060, China; 3 School of Science, Faculty of Health and Environmental Sciences, Auckland University of Technology, Auckland 1142, New Zealand; 4 College of Food Science and Technology, Nanchang University, Nanchang 330031, China; 5 Maurice Wilkins Centre for Molecular Biodiscovery, Auckland 1142, New Zealand

**Keywords:** alginate, food hydrocolloid, food film packaging, health effects

## Abstract

Alginate is an acidic polysaccharide mainly extracted from kelp or sargassum, which comprises 40% of the dry weight of algae. It is a linear polymer consisting of β-D-mannuronic acid (M) and α-L-guluronic acid (G) with 1,4-glycosidic linkages, possessing various applications in the food and nutraceutical industries due to its unique physicochemical properties and health benefits. Additionally, alginate is able to form a gel matrix in the presence of Ca^2+^ ions. Alginate properties also affect its gelation, including its structure and experimental conditions such as pH, temperature, crosslinker concentration, residence time and ionic strength. These features of this polysaccharide have been widely used in the food industry, including in food gels, controlled-release systems and film packaging. This review comprehensively covers the analysis of alginate and discussed the potential applications of alginate in the food industry and nutraceuticals.

## 1. Introduction

**Figure 1:**
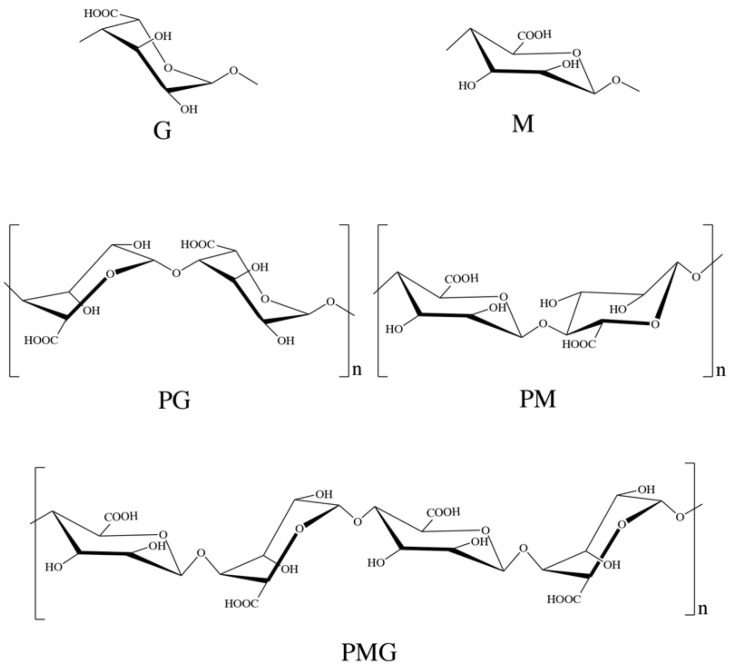
Structure of alginate.

Alginate, discovered in the 1880s [1], is a linear polysaccharide produced by brown algae and bacteria [2,3]. It is not only a biopolymer but also a polyelectrolyte that is considered to be non-toxic, biocompatible, biodegradable, and non-immunogenic. Alginate is an anionic copolymer, and its structure is depicted in Figure 1, which includes β-D-mannuronic acid (M) and α-L-guluronic acid (G) linked by 1,4-glycosidic bonds [4,5]. It is arranged in an irregular block-wise pattern of varying proportions of GG, MG and MM blocks. The physicochemical properties of alginate are critically affected by the M/G ratio and the length of each block [6]. The MM blocks form a β-1,4-glycosidic bond, making the M block section presents a linear flexible structure, while GG blocks have α-1,4-glycosidic bonds, introducing space steric hindrance around the carboxyl. For this reason, the G-block segment provides a folded and rigid structural conformation that is responsible for the significant stiffness of the molecular chain [6].

Alginate is known to form hydrogels in the presence of divalent cations. With divalent cations, especially Ca^2+^, alginate can form a gel matrix. These features enable this polysaccharide to be used to control-release certain food ingredients, bioactive compounds and pharmaceutical materials for various products [7].

Alginate is a general name for water-soluble alkali metal salts [8]. Alginate is a major constituent of the brown algae (mainly sargassum algae and kelp) cell wall. It takes about 40% of the dry weight of algae [6]. Alginate is mainly extracted based on its high solubility in alkaline solution and low solubility in water. Sodium carbonate solution is used to dissolve alginate from the algae cell wall, and then precipitate it out via adjustment of the pH value with acid [9]. The main species of algae used for alginate extraction are *Laminaria hyperborean*, *Macrocystis pyrifera*, *Laminaria digitata*, *Ascophyllum nodosum*, *Sargassum* spp., *Laminaria japonica*, *Ecklonia maxima* and *Lessonia nigrescens* [10]. Since there is no modification group such as sulfate in brown alginate, there is no need to consider the problem of modification groups dropping in the process of acid precipitation [8,9].

Recently, the development of an alginate delivery system and its other application in the food industry has attracted more and more interest. This review briefly introduces the structure and functional properties of alginate, including food gels, controlled-release systems, film packaging and potential application as functional foods. The purpose of this paper is to provide reference for the design and manufacture of alginate-based nutraceutical delivery systems and their direct application in health care products in the future.

## 2. Structure, Derivatization and Analysis

Alginate structure is illustrated in Figure 1 where there are blocks of residues along the chain. There are homopolymeric regions of polyguluronic acid (PG) and polymannuronic acid (PM), interspaced with hetero-polymeric regions with PM-PG residue mixtures (PMG). Different brown algae produce different proportion and sequence of M and G residues, which influence the molecular weight and physical properties of alginate [7]. These structures in alginate are the result of a unique biosynthetic pathway in which G residues are generated from preformed polymers of mannuronic acid by a family of isoenzymes with C-5 epimerase activity [11,12,13]. PM and PG can be separated by hydrolyzing alginate with hydrochloric acid (HCl) at pH 2.85. At this pH, the soluble portion contains 80–90% M residues, and the insoluble precipitate contains 80–90% G residues [14]. The content of G in Sargassum is higher, while the content of M in kelp is higher. The M/G ratio in alginate is not fixed. Even for the same seaweed, the proportion will change with different growth years, picking seasons and locations [15].

For the primary structure, M and G differ only in the position of the carboxyl group on the C5 site, but just because of this small difference, they have significant differences in spatial structure and physical properties. The spatial structure of PG shows that monosaccharide units are in the 1C chair conformation and are stabilized by hydrogen bonds between intramolecular O2 and O6, while PM is a boat conformation and stabilized by hydrogen bonds between intramolecular O2 and O5, resulting in folded and rigid conformations of PG and a linear, flexible and flat conformation of PM [16]. Therefore, the high G content provides higher strength for alginate than high M content [17]. Additionally, these structural differences lead to great differences in the acid hydrolysis resistance of PM, PG and PMG fragments. PMG is easily hydrolyzed, while PM and PG are not easily hydrolyzed, and the acid hydrolysis resistance of PG is obviously stronger than that of PM [14].

Alginate is widely used in industry because of its ability to gel with calcium ions; however, this property is also strongly influenced by its uronic acid composition, i.e., M/G ratio [18]. The M/G ratio was originally determined using two-step hydrolysis of sulfuric acid at different concentrations and paper chromatography to separate M and G. Haug et al. improved this method, which was to separate each fragment by heterogeneous partial acid hydrolysis and fractional precipitation and then determine the uronic acid composition of each fragment by complete acid hydrolysis [14]. However, identifying blocks in this way is laborious and time-consuming, requiring a large amount of material [19]. Based on the carbazole reaction, which can give very different color intensities for mannuronic and guluronic acids [20], Knutson et al. established a method to determine the M/G ratio under two different reaction conditions, and this method worked well on mixtures of mannuronic and guluronic acids [21,22]. However, in the specific application of M/G ratio detection of alginate, the content of G is consistently overestimated, which may be because carbazole reagents react differently from acids in polymer and monomer forms [18]. The determination of the M/G ratio by nuclear magnetic resonance (NMR) spectroscopy after alginate hydrolysis has improved substantially in terms of time and material requirements [18,23,24], but it is still not fully suitable for routine screening of large numbers of samples and spectra often needed to be acquired at high temperature to decrease the viscosity of the alginate solution [25,26]. Morris et al. found that the circular dichroism (CD) spectra of alginates showed a peak at 200 nm, and a trough at 215 nm, whose relative magnitudes vary systematically with composition [19]. Based on this, they established a simple equation to determine the relative amounts of M and G from the observed ratio of peak height to trough depth. This method can obtain a reliable compositive estimate from 1 mg alginate, and the results obtained are consistent with those obtained by the hydrolysis and NMR analysis of the same sample [19]. For high sensitivity analysis of the M/G ratio of alginate and its derivatives, which were deeply hydrolyzed and then performed on high-performance liquid chromatography (HPLC) [27], anion-exchange liquid chromatography (AELC) [28], capillary electrophoresis (CE) [29], gas chromatography (GC) [30] and high-performance anion-exchange chromatography (HPAEC) combined with using pulsed amperometric detection for separation-based analysis were performed [26]. These methods, however, need to hydrolyze the polysaccharide samples and cannot take into account the recovery, or require derivatization after hydrolysis of polysaccharides to monosaccharides.

Alginate is a kind of hydrophilic polysaccharide, which can be modified into amphiphilic or hydrophobic molecules by derivation of hydroxyl and carboxyl groups. In general, the derivatization of alginate occurs at the -OH position (C-2 and C-3) or -COOH (C-6) [17]. The modification of the carboxyl group in the C6 position of alginate is usually through esterification or amino reaction to introduce long alkyl groups or fatty acids, which makes alginate become amphiphilic molecules [31,32]. On the other hand, the hydroxyl group can be modified by sulfation [33,34], phosphorylation [35,36] and selenization [37,38,39,40] to improve the bioactivity of alginate. Due to the distribution of a large number of hydroxyl and carboxyl groups in the main chain of alginate, it can be easily chemically modified to improve its characteristics. Although a lot of work has been done on the synthesis of alginate derivatives, there are still many potential pathways to be studied. Meanwhile, the application of sodium alginate derivatives in various fields still has great prospects.

## 3. Traditional Application in Food

### 3.1. Hydrocolloidal Gel

Hydrocolloid gel particles have potential application value in the food, chemical and pharmaceutical industries. Alginate gel particles with the advantages of good biocompatibility, non-toxic, biodegradable, low price and simple production, are one of the most widely used gel particles at present [7]. Also, they are particularly valuable for encapsulating applications, protecting cells, DNA, nutrients and microorganisms, and also enabling the slow release of flavors, minerals and drugs by encapsulating in gel particles [41]. Importantly, it has been determined that the compound loaded in Ca-alginate gel particles does not adversely affect its flavor release during consumption [42]. The application and limitation of alginate-based hydrocolloidal gels was summarized in Table 1.

#### 3.1.1. Ionic Gel

Alginate has the ability to form an ionic gel in the presence of multivalent cations, which is widely utilized in the encapsulation of active substances in the food industry. The binding of alginate with divalent cations has high selectivity, and the affinity of alginate to cations is Mn < Zn, Ni, Co < Fe < Ca < Sr < Ba < Cd < Cu < Pb, which depends directly on G content in the alginate [43]. This is because in the formation of gels, it is mainly G that binds to divalent cations, and the mechanism is the dimerization of G, resulting in a tightly bound polymer whose structure forms an “egg-box” shaped junction region [7]. Alginate with high G content can format gels which are strong and brittle, with good thermal stability, while alginate gels with high M content are weak and more elastic, with good freeze-thaw properties [7,17,44]. In practical applications, considering toxicity and other reasons, the calcium ion is the most widely used ion to prepare the alginate gel (Ca-alginate gel).

The methods of Ca-alginate gel particles formation include external gelation or internal gelation. The main difference is that the Ca^2+^ is introduced into alginate polymers in different ways [7]. In the internal gelation, alginate exposure to Ca^2+^ was controlled to achieve uniform distribution of alginate in hydrogel. Gelation occurs simultaneously anywhere in the alginate solution, resulting in a uniform hydrogel structure. First, insoluble calcium salt such as CaCO_3_ or CaSO_4_ are added to a solution of sodium alginate and extruded into oil [45]. Acid is then added to acidify the mixture, releasing Ca^2+^ from these compounds. This process can be achieved either immediately, by direct addition of glacial acetic acid [45,46], or in a controlled fashion using D-glucono-d-lactone [46,47,48]. In the external gelation method, Ca^2+^ is diffused from a region of higher concentration into the interior of alginate. In the outermost layer of the hydrogel, the concentration of Ca^2+^ is high, which enables the alginate to gel rapidly. Ca-alginate gel produced by this method is inhomogeneous, with high Ca^2+^ and alginate gradients near the gel surface that decrease as the core is approached [7,46].

Puguan et al. compared the structure, physical and chemical properties and diffusion behavior characterization of calcium alginate gel prepared by these two methods in detail [49]. The results showed that the Ca-alginate gel particles synthesized by the internal gelation method have looser structure and larger pore size than those synthesized by the external gelation method. As a result, vitamin B12 diffused faster in the gel particles prepared by the internal gelation method [49]. Additionally, it was found that (I) the Ca^2+^ concentration was the decisive factor in gel formation, (II) the loss of weight and volume were effective because of water removal, (III) Na^+^ acted as the competitor with Ca^2+^ and (IV) the pH values controlled the gel formation by regulating alginate dissociation and calcium complexation in Ca-alginate gel particles prepared by external gelation method [49]. Temperature also has an influence on gel formation. Jeong et al. reported that lowering the gel temperature can slow down the diffusion rate of Ca^2+^ in the Ca-alginate gel particles, making the internal structure of Ca-alginate gel particles more regular and improving the rupture strength of the gels [50]. In the thermal treatments, the water loss promoted the formation of a dense, porous structure in the gels, which would improve the fracture strength of the Ca-alginate gel particles [51]. However, alginate gel particles tend to harden after drying, which limits their application. A recent study found that the addition of glycerol can stabilize the gel network structure and improve the tolerance of gel particles by hydrogen bonding with alginate [52]. However, a major limitation of calcium-alginate gels is that they become unstable in the presence of calcium chelators such as citrate, phosphate, carbonate and lactate [7].

Diffusion characteristics studied showed that the concentration of alginate and Ca^2+^ did not affect the diffusion of solutes into Ca-alginate gel particles with molecular weight < 20,000 Da [53]. However, the study on diffusion coefficient during the release of vitamin B12 showed that the diffusion coefficient of vitamin B12 decreased slightly with the increase of alginate concentration, and the lower the calcium concentration, the higher the release of vitamin B12 [53]. Lopez-Sanchez et al. reported that the Ca-alginate gel particles with high concentrations sugar (glucose:fructose, >60%) were characterized by less connectivity and open network aggregation chains in gastric fluid. The reason might be that the presence of high sugars as co-solute results in less alginate chain extension and reduced connectivity of the calcium alginate network. This swelling and shrinkage of gels influenced the release of sugars from Ca-alginate gel particles. While the sugar content did not exceed 30% (wt), the release mechanism from Ca-alginate gel particles was similar to the diffusion driving mechanism, suggesting that Ca-alginate gel particles can be used as a carrier of low molecular weight sugars (sugar < 30%) without blocking the release of sugars in the digestive tract [54].

In food applications, Ca-alginate gel particles used to encapsulate plant polyphenols could improve the functionality and stability of polyphenols in food products. As reported by Stojanovic et al., Ca-alginate gel particles could effectively prevent the degradation of active substances by encapsulating the *Thymus serpyllum* L. aqueous extracts, and there was no chemical interaction between the active substances and the alginate. In addition, the encapsulation efficiency could be up to 80% [55]. Similar studies on the encapsulation of lemon balm extracts by Ca-alginate gel particles showed that Ca-alginate gel particles could maintain the antioxidant activity of lemon balm extracts, and there was no chemical interaction between lemon balm extracts and the alginate, indicating the fitness of alginate for loading natural antioxidants [56]. In order to achieve efficient and sustained release of catechin, Kim et al. prepared Ca-alginate gel particles loaded with catechin by emulsifying gel method using sunflower seed oil as raw material. The continuous release of catechins under acidic conditions was determined, suggesting that Ca-alginate gel particles by emulsion gelation method are an effective catechin transport system [57]. However, this only allows catechins to be released mostly in the stomach and does not allow catechins to enter the gut. Ca-alginate gel particles also can be used to encapsulate probiotics. Ca-alginate gel particles containing *Lactobacillus rhamnosus* and *Lactobacillus acidophilus* were prepared by a novel impinging aerosols method, with the particle size below the limits of sensory detection. This particle could provide some protection for these probiotics in high acid and bile environments [58]. On the other hand, Petzold et al. prepared the Ca-alginate gel particles loaded with liquid smoke flavoring using the dripping method, and the load capacity reached above 96%. Importantly, it could release several volatile compounds while the particles heated [59]. Ca-alginate gel particles also could be used to immobilize D-limonene, the major flavor compound of citrus oil, to maintain the thermal stability of D-limonene [42].

In foods, in order to minimize the powdery or grainy sensation found in foods such as yogurt and ice cream, the average diameter of gels used should ideally not exceed 30 μm [60]. This allows food manufacturers to increase the number of bioactive ingredients in their products while maintaining their original taste and texture. Therefore, in food production, the size of most reports about Ca-alginate gel particles is less than 30 μm.

#### 3.1.2. Acidic Gel

Since the dissociation constant (pKa) of M and G residues are 3.38 and 3.65, respectively, this causes alginate to be negatively charged over a wide pH range. When the pH value of the solution falls below the pKa of the residues, alginate in the solution could form gels spontaneously [61]. Two forms of colloid can be formed by adjusting the rate of pH value reduction in alginate solution. When the pH value of the solution decreases rapidly, alginate molecules will precipitate in the form of aggregation, while when the pH value decreases slowly and steadily, continuous alginate bulk gel will be formed [62,63]. Unlike the ionic gel, the acid gel of alginate is steadied by hydrogen bonds, and M residues are also involved in the formation of gels [64]. The similarity is that gel strength is related to G residue content in the polymer chain [63]. The low pH required for colloid formation makes the acid gel of alginate difficult to use in the food field [7]. However, it can be used as an antacid to relieve gastric reflux and heartburn [65,66].

#### 3.1.3. New Emulsion Gel

Ca-alginate gels are pH sensitive and are often used to construct core-shell structures, but they are very unstable in the gastrointestinal tract. Therefore, using Ca-alginate gel as a delivery system to load nutrients or active substances could cause most of the nutrients or active substances to be released in the stomach, exposing them to strong acid conditions and causing damage. This delivery system cannot guarantee the smooth passage of the nutrients or active substances loaded in the gels into the gut. At present, researchers have not only used alginate to prepare gels but also introduced some new substances, including but not limited to protein and polysaccharides, to enhance colloidal properties. Lin et al. introduced the soy protein isolate into the Ca-alginate gels-based emulsion and found that the soy protein isolate could improve the properties of the emulsion, including morphological properties, shrinkage, water loss and elasticity [67]. They also found that adding a low concentration of soy protein isolate-stabilized emulsions into alginate solutions could produce a more stable emulsion than adding alginate solutions into soy protein isolate-stabilized emulsions with mild stirring [68]. A similar study showed that casein and whey protein isolate also could improve the properties of Ca-alginate-based emulsion gels [48,69]. However, the viscosity of the Ca-alginate gels-based emulsion after introducing whey protein isolate was higher than that of the emulsion after introducing soy protein isolate. The particle size distribution of droplets of the emulsion introduced whey protein isolate was smaller than that of emulsion introduced soy protein isolate, as well as the flocculation phenomenon [70]. Similar to the introduction of soy protein isolate, the introduction of whey protein isolate also can prevent the emulsion from losing water during the gel process [67,70]. Importantly, the presence of whey protein isolates and soy protein isolate resulted in a higher shrinkage rate and Young’s modulus change rate of emulsions during in vitro gastric digestion, and delayed lycopene release of emulsions during in vitro intestinal digestion [70]. A new core-shell structure (sporopollenin exine capsules as the core and Ca-alginate (Alg)/carboxymethylpachymaran (CMP) gel as the shell) was developed to protect probiotics, both to improve the storage and lyophilization stability of probiotics and to achieve sustained release in the gastrointestinal tract [71].

Furthermore, the induction of the protein and other substances into the alginate-based emulsion gels can improve the stability of an active substance or nutrient loaded in the gels. Chen et al. reported that the emulsion gels can protect both lipophilic and hydrophilic bioactive substances, while those bioactive substances coated with oil droplets are more likely to be retained during heating [47]. Simultaneous encapsulation of two bioactive substances (Epigallocatechin gallate and β-carotene) in whey protein isolate/ Ca-alginate-based emulsion gels can produce synergistic effects and improve their chemical stability [47]. A double cross-linked emulsion gel with a dense mesh structure and high viscoelasticity was prepared by cross-linking zein with transglutaminase and alginate with the calcium ion. By comparing the effects of gels on the photostability and bio-accessibility of co-loaded polyphenols (curcumin and resveratrol), it was found that double crosslinked emulsion gels had higher photostability and bio-accessibility than single crosslinked emulsion gels [72].

Ca-alginate gel particles can also be used to prepare low-fat mayonnaise and other similar emulsions [73]. Yang et al. prepared a type of thixotropic and viscoelastic emulsion gel by using alginate and konjac glucoman, which showed good thermal and freeze-thaw stability. Moreover, no oil droplets coalescence was observed after the emulsion was heated at 100 °C for 30 min or frozen at −18 °C for 24 h. These results suggested that konjac glucoman/Ca-alginate gel emulsions system is expected to provide a template for the design of low-fat mayonnaise food emulsions [74]. Yang et al. also prepared an oil-in-water emulsions through electrostatic complexation between alginate and egg yolk proteins at acidic pH (<5.0), and the emulsion gel products showed good viscoelasticity, thixotropy and were comparable to 75% oil full fat mayonnaise products. To obtain a good sensory profile, vinegar was used for condiments added into the products [75]. These studies may contribute to the decrease in overconsumption of fatty foods in humans and thus reduce the incidence of chronic diseases in humans.

### 3.2. Film Packaging

Environmentally safe and biodegradable natural polysaccharide materials are a new type of film packaging, mostly made from extracts of by-products of fruit and vegetable processing, which not only maximizes the value of fruit and vegetables, but also reduces waste and improves the environment [76]. Alginate has unique colloidal properties, including thickening, stabilization, suspension, film formation, gel formation and emulsion stabilization. This allows films made from alginate to be strong and resistant to oil and grease [77], but to have poor water resistance due to their hydrophilicity [78]. Therefore, alginate is generally mixed with other biopolymers to improve the mechanical properties of the films. The application and limitation of alginate-based film was summarized in Table 1. Ismillayli et al. reported that the carboxyl group of alginates and the ammonium group of chitosans can have electrostatic interactions. Under the same thickness, the tensile strength and resistance to pH changes of the alginate-chitosan film were higher than that of natural alginate film and chitosan film. In addition, the alginate-chitosan membrane has good antibacterial potential against Staphylococcus aureus and *Escherichia coli* [79]. Reyes-Avalos et al. reported that alginate-chitosan coating is an excellent post-harvest technology for preserving not only the organoleptic and sensory properties of figs during cryopreservation but also their bioactive constituents by modifying the internal atmosphere of figs [80,81].

The complex film prepared by alginate only or with other polymers combined with some substances with antibacterial and antioxidant activities not only has good mechanical properties but also has some special bio-activities. For example, Gelatin–alginate film containing 1.5% oregano essential oil could effectively delay bacterial growth on rainbow trout (Oncorhynchus mykiss) slices, including lactic acid bacteria, Pseudomonas spp. and Enterobacteriaceae [82]. While the gelatin-alginate film was prepared by incorporating tea polyphenols, not only were the mechanical properties of the films improved, but their antioxidant activity also improved [83].

Addition of vitamin C to the alginate-based edible film decreased the tensile strength of the film, but it made the film more stable, and it could be stored at refrigeration in the dark for up to five months [84]. A new polysaccharide composite film packaging with good tensile strength and elongation at break was prepared from citrus pectin and alginate. After being crosslinked with calcium chloride, the water solubility of the film decreased, and the thermal stability increased. The addition of pterostilbene as an antioxidant reduced the values of tensile strength and elongation at break, but gave better water resistance and oxidation resistance, showing that this film could be utilized as an excellent antioxidant packaging material in fruit and vegetable preservation [76]. The addition of epigallocatechin gallate into alginate and carboxymethyl cellulose prepared edible films could improve the tensile strength of the edible films and reduce their elongation at break, and also showed strong antioxidant activity in fatty foods [85].

Alginate films incorporated with lemon-grass oil and glycerol, which acted as a natural antimicrobial agent and plasticizer, respectively, are also effective in inhibiting the growth of *Escherichia coli* [78]. Adding AgNPs to alginate-based edible film improved the tensile strength and elongation at break, and the growth inhibition rate of alginate-based edible film was higher than 79% in all strains [86]. Adding hawthorn berry (Crataegus pinnatifida) extract [87], mulberry (Morus australis) leaf extract [88] or essential oils [89] to alginate-based edible film made not only similar mechanical property improvements and antibacterial effects but also can improve the sensory sensation of foods [90]. In films prepared from alginate/pullulan and capsaicin, with an increase of capsaicin content, the light transmittance, elongation at break and moisture content of the films decreased, while the tensile strength, permeability and surface contact angle increased. In addition, the film has good antibacterial performance against *Escherichia coli* and Staphylococcus aureus and has achieved good results in apple protection [91].

Alginate is widely found in seaweed, so the alginate-based film has the advantage of low cost. Although the alginate-based film has poor water resistance, its compatibility with other polysaccharides can ameliorate this shortcoming. Furthermore, the addition of some active substances into the film endows the film with good antibacterial and antioxidant activities, thus extending the shelf life of food. Up until now, there have been few studies on the application of AOS in the preparation of edible films, possibly due to poor colloidal properties. However, AOS have good biological activity and may play certain functions in other polysaccharide-based films.

**Table 1:** The type of alginate-based materials.

Alginate-Based Materials		Application	Limitation	Reference
Hydrocolloidal gel	Ionic gel	Encapsulation of active substance in the food industry	1. Tend to harden after drying 2. Unstable in the presence of calcium chelators	[42,43,44,45,46,47,48,49,50,51,52,53,54,55,56,57,58,59]
	Acidic gel	An antacid to relieve gastric reflux heartburn	Low pH required for colloid formation	[7,65,66]
	Emulsion gel	1. Encapsulation of active substance. 2. Low-fat mayonnaise and other similar emulsions	Not mentioned	[67,68,69,70,71,72,73,74,75]
Film	Made from alginate only	Packaging	Poor water resistance	[77,78]
	Made from alginate and other biopolymers	Packaging	Not mentioned	[78,79,80,81,82,83,84,85,86,87,88,89,90,91]

## 4. Potential Application as Functional Foods of Alginate

### 4.1. Reducing Obesity and Resistance to Diabetes

Obesity has become a pandemic affecting a third of the world’s population, which brings high risk of type 2 diabetes to those obese people [92]. Currently, there are not many effective and safe anti-obesity drugs on the market. An emerging trend is to use natural ingredients from food to combat obesity. Alginate has been developed as a food supplement for energy restriction, which can improve weight loss in obese subjects. This has been shown in a 12-week dietary intervention project [93]. It might be that alginate can form both acid and ionic gels in the stomach, leading to a decrease in the activity of digestive enzymes such as pancreatic lipase and subsequent satiation [94,95,96]. Similarly, Guo et al. reported that the calcium carbonate-containing sodium alginate system formed a gel in stomach conditions, and the formation of the gel lowered the dextrin and whey protein isolate (WPI) hydrolysis rate in vitro [97]. They also found that long-term feeding containing sodium alginate in the diet could reduce food intake, body weight, apparent protein digestibility and blood glucose in rats, indicating that alginate could potentially be effective in the treatment of obesity [97]. However, another study has reported that sustained consumption of alginate over a short period of time (10 days) has been proven to have no effect on gastric motor functions, satiation, appetite or gut incorporation, raising doubt on whether short-term alginate treatment is effective in weight loss [98]. In a high-fat diet with streptozotocin (STZ) injection-induced type 2 diabetic mice, alginate from *Sargassum fusiforme* can effectively reduce blood glucose, TG and TC, and can increase HDL-c and improve glucose tolerance. Furthermore, administering alginate to diabetic mice has a moderate effect on adipose hepatic, skeletal muscle and heart tissues’ pathological changes. It also can diminish oxidative stress [99].

### 4.2. Regulation of Gut Microbiota

It has been shown that gut microbes can affect many diseases, including food allergies [100], AD [101], and obesity [102,103]. There is much evidence that alginate and AOS interact with intestinal microbes, which may affect a number of diseases. As mentioned above, oral alginate can restore the ovalbumin-induced gut microbiota disorder in a mouse model of egg allergy, recovering the richness and diversity of gut microbial groups [104]. Alginate from *Sargassum fusiforme* can significantly increase benign bacteria including Lactobacillus, Bacteroides, Akkermansia Alloprevotella, Weissella and Enterorhabdus, and significantly decrease harmful bacteria such as Turicibacter and Helicobacter in a type 2 diabetic mouse model. Meanwhile, alginate can significantly decrease branched-chain amino acids (BCAAs) and aromatic amino acids (AAAs) in the colons of type 2 diabetic mice, indicating a positive benefit of alginate in type 2 diabetes [99]. Huang et al. reported that alginate extracted from Laminaria increased the abundance of beneficial bacteria but decreased pathogenic bacteria in the intestine of immunosuppressed BALB/c mice induced by cyclophosphamide. Additionally, alginate treatment can reverse intestinal mucosal injury and increase intestinal permeability by upregulating the expression of tight junction proteins, indicating that alginate may be able to enhance immunity [105]. Ejima found that diets with alginate could suppress HFD-induced metabolic syndrome (MetS) via an effect on the gut microbiota, including changing the gut microbiota composition and increasing the abundance of Bacteroides [106]. Similarly, alginate from *Laminaria japonica* could also increase the abundance of Bacteroides after fermenting with fresh fecal samples from healthy volunteers [107]. Al-Najjar et al. found that when Wistar rats were fed calcium-crosslinked alginate aerogel for 14 days at a dose of 250 mg/day, the Clostridia and Bacteroides groups increased and continued to increase after aerogel feeding. Other gut bacteria groups, including Erysipelotrichia and Candidatus saccharibacteria, increased during the aerogel administration, and then decreased one month afterwards [108].

Generally, alginate is not digested in the upper digestive tract. When it reaches the colon, it can be used by gut microbes, which digest it into short chain fatty acids (SCFAs), which promotes the growth of beneficial bacteria and inhibit harmful bacterial growth [109]. In addition, in obesity and type 2 diabetes, SCFAs in the colon can maintain the health of the host and prevent colonic diseases [110].

### 4.3. Immunomodulatory and Antitumor Activities

Receptors on surface of innate immune cells are important for recognizing pathogens and initiating immune responses. They play essential roles in the regulation and activation of complement and phagocytosis, initiation of pro-inflammatory signaling pathways, and induction of apoptosis [111]. Kurachi et al. compared the abilities of alginate polymers with different Mw and M/G rations on tumor necrosis factor (TNF)-α production in RAW264.7 cells, confirming that alginate treatment could induce TNF-α release in RAW264.7 cells [112]. Yang et al. found that alginate caused innate immune responses in macrophage-like cells (RAW264.7 cells), inducing the production of cytokines, such as interleukin (IL)-1β, IL-6, IL-12 and TNF-α with time and dose-dependence, through nuclear factor kappa-light-chain-enhancer of activated B cells (NF-κB) signaling pathway activation [113]. Further study showed that alginate can not only activate the NF-κB signaling pathway but also the p38 mitogen-activated protein kinase (MAPK) signaling pathway by Toll-like receptor 4 (TLR4) activation in RAW264.7 cells, and then enhance the intracellular phagocytosis of gold nanoparticles, fluorescent microspheres and immunoglobulin G (IgG)-opsonized Staphylococcus aureus [114]. Alginate also can attenuate the systemic anaphylaxis response in compound 48/80-induced Wistar rats via the suppression of NF-κB activation [115]. In addition, the alginate aqueous solution (Mw = 108 kDa) also exhibited non-Newtonian characteristics, including viscoelasticity and shear-thinning behavior, which may be a significant factor affecting the ability of the gastrointestinal tract to contact and take in ovalbumin, the main allergen that causes egg allergy. In ovalbumin-induced mouse models of egg allergy, oral alginate aqueous solutions can effectively attenuate the occurrence of allergic reactions, decrease the histamine IgE and IL-4 levels in serum, increase the level of IFN-γ in serum, increase the number of Treg cells in spleen tissues and inhibit differentiation of T-helper type 0 (Th0) cells into Th2 cells [116]. Further research showed that oral alginate can restore the ovalbumin-induced gut microbiota disorder, recovering the richness and diversity of Alloprevotella, Bacteroides, Parabacteroides and Rikenellaceae_RC9_gut_group [104].

Cancer is the leading cause of death in economically developed countries and the second most common cause of death in developing countries. More than 30 years ago, Fujihara et al. reported the alginate showed antitumor activity against various murine tumors, such as Sarcoma-180 and Ehrlich ascites carcinoma tumors [117] and found that the higher content of M block in alginate may correlate with higher antitumor activity [118] and that the antitumor activity of alginate could be improved by adding Ca^2+^ [119].

## 5. Conclusions and Prospects

**Figure 2:**
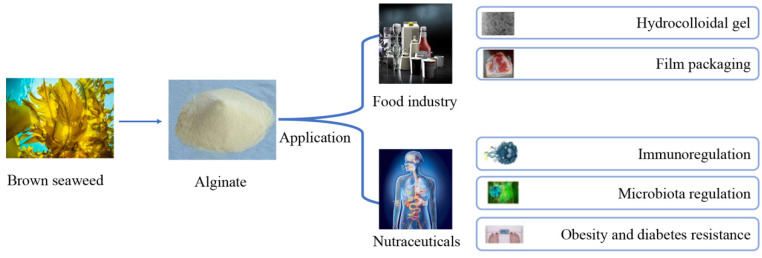
The application of alginate in the food and nutraceutical field.

Alginate is a natural and safe food additive. Compared with other seaweed polysaccharides, alginate has excellent functional properties such as ion cross-linking, pH sensitivity, biocompatibility and biodegradability, which has been widely applied in the food and nutraceutical fields (Figure 2). Alginate is also the only polysaccharide that naturally contains carboxyl groups in each constituent residue. It can be used to produce food colloids and food films. In addition, it has a variety of good biological activities, providing opportunities for it to be developed into functional foods and nutraceuticals. Although research progressions are made toward the production and application of alginate in the food and nutraceutical industries, more investigations are still required to improve our understanding of its bioactivities and potential usages. The improved analysis methods and safety knowledge are the foundation for its development in functional foods or nutraceuticals. Further research in the above area will certainly provide an improved basis for the future production and application of alginate.

## References

[1] Stanford E.C.C. (1883). On algin: A new substance obtained from some of the commoner species of marine algae. Chem. News.

[2] Linker A., Jones R.S. (1964). A polysaccharide resembling alginic acid from a Pseudomonas micro-organism. Nature.

[3] Linker A., Jones R.S. (1966). A new polysaccharide resembling alginic acid isolated from pseudomonads. J. Biol. Chem..

[4] Brownlee I.A., Allen A., Pearson J.P., Dettmar P.W., Havler M.E., Atherton M.R., Onsøyen E. (2005). Alginate as a Source of Dietary Fiber. Crit. Rev. Food Sci. Nutr..

[5] Bi D., Yang X., Lu J., Xu X. (2022). Preparation and potential applications of alginate oligosaccharides. Crit. Rev. Food Sci. Nutr..

[6] Flórez-Fernández N., Torres M.D., González-Muñoz M.J., Domínguez H. (2019). Recovery of bioactive and gelling extracts from edible brown seaweed *Laminaria ochroleuca* by non-isothermal autohydrolysis. Food Chem..

[7] Ching S.H., Bansal N., Bhandari B. (2017). Alginate gel particles—A review of production techniques and physical properties. Crit. Rev. Food Sci. Nutr..

[8] Onsøyen E. (1997). Alginates. Thickening and Gelling Agents for Food.

[9] Haug A. (1964). Composition and properties of alginates. Report No. 30.

[10] Liu J., Yang S., Li X., Yan Q., Reaney M.J.T., Jiang Z. (2019). Alginate Oligosaccharides: Production, Biological Activities, and Potential Applications. Compr. Rev. Food Sci. Food Saf..

[11] Larsen B., Haug A. (1971). Biosynthesis of alginate: Part I. Composition and structure of alginate produced by *Azotobacter vinelandii* (Lipman). Carbohydr. Res..

[12] Haug A., Larsen B. (1971). Biosynthesis of alginate: Part II. Polymannuronic acid C-5-epimerase from *Azotobacter vinelandii* (Lipman). Carbohydr. Res..

[13] Larsen B., Haug A. (1971). Biosynthesis of alginate: Part III. Tritium incorporation with polymannuronic acid 5-epimerase from *Azotobacter vinelandii*. Carbohydr. Res..

[14] Haug A., Larsen B., Smidsrød O., Eriksson G., Blinc R., Pausak S., Ehrenberg L., Dumanović J. (1967). Studies on the Sequence of Uronic Acid Residues in Alginic Acid. Acta Chem. Scand..

[15] Llanes F., Ryan D.H., Marchessault R.H. (2000). Magnetic nanostructured composites using alginates of different M/G ratios as polymeric matrix. Int. J. Biol. Macromol..

[16] Atkins E., Nieduszynski I., Mackie W., Parker K., Smolko E. (1973). Structural components of alginic acid. II. The crystalline structure of poly-α-L-guluronic acid. Results of X-ray diffraction and polarized infrared studies. Biopolym. Orig. Res. Biomol..

[17] Xu X., Bi D., Wan M. (2016). Characterization and Immunological Evaluation of Low-Molecular-Weight Alginate Derivatives. Curr. Top. Med. Chem..

[18] Penman A., Sanderson G.R. (1972). A method for the determination of uronic acid sequence in alginates. Carbohydr. Res..

[19] Morris E.R., Rees D.A., Thom D. (1980). Characterisation of alginate composition and block-structure by circular dichroism. Carbohydr. Res..

[20] Dische Z. (1947). A new specific color reaction of hexuronic acids. J. Biol. Chem..

[21] Knutson C.A., Jeanes A. (1968). A new modification of the carbazole analysis: Application to heteropolysaccharides. Anal. Biochem..

[22] Knutson C.A., Jeanes A. (1968). Determination of the composition of uronic acid mixtures. Anal. Biochem..

[23] Grasdalen H., Larsen B., Smidsrød O. (1979). A p.m.r. study of the composition and sequence of uronate residues in alginates. Carbohydr. Res..

[24] Grasdalen H., Larsen B., Smisrod O. (1981). 13C-NMR studies of monomeric composition and sequence in alginate. Carbohydr. Res..

[25] Rahelivao M.P., Andriamanantoanina H., Heyraud A., Rinaudo M. (2013). Structure and properties of three alginates from Madagascar seacoast algae. Food Hydrocoll..

[26] Lu J., Yang H., Hao J., Wu C., Liu L., Xu N., Linhardt R.J., Zhang Z. (2015). Impact of hydrolysis conditions on the detection of mannuronic to guluronic acid ratio in alginate and its derivatives. Carbohydr. Polym..

[27] Voragen A., Schols H., De Vries J., Pilnik W. (1982). High-performance liquid chromatographic analysis of uronic acids and oli-gogalacturonic acids. J. Chromatogr. A.

[28] Gacesa P., Squire A., Winterburn P.J. (1983). The determination of the uronic acid composition of alginates by anion-exchange liquid chromatography. Carbohydr. Res..

[29] Guttman A. (1997). Analysis of monosaccharide composition by capillary electrophoresis. J. Chromatogr. A.

[30] Rumpel C., Dignac M.-F. (2006). Gas chromatographic analysis of monosaccharides in a forest soil profile: Analysis by gas chro-matography after trifluoroacetic acid hydrolysis and reduction–acetylation. Soil Biol. Biochem..

[31] Yang J.-S., Xie Y.-J., He W. (2011). Research progress on chemical modification of alginate: A review. Carbohydr. Polym..

[32] Pawar S.N., Edgar K.J. (2013). Alginate esters via chemoselective carboxyl group modification. Carbohydr. Polym..

[33] Gionet-Gonzales M., Casella A., Diloretto D., Ginnell C., Griffin K.H., Bigot A., Leach J.K. (2021). Sulfated alginate hydrogels prolong the therapeutic potential of MSC spheroids by sequestering the secretome. Adv. Healthc. Mater..

[34] Park J., Lee S.J., Lee H., Park S.A., Lee J.Y. (2018). Three dimensional cell printing with sulfated alginate for improved bone morphogenetic protein-2 delivery and osteogenesis in bone tissue engineering. Carbohydr. Polym..

[35] Pallmann J., Ren Y.L., Mahltig B., Huo T.G. (2019). Phosphorylated sodium alginate/APP/DPER intumescent flame retardant used for polypropylene. J. Appl. Polym. Sci..

[36] Putri A.P., Picchioni F., Harjanto S., Chalid M. (2021). Alginate Modification and Lectin-Conjugation Approach to Synthesize the Mucoadhesive Matrix. Appl. Sci..

[37] Bi D., Lai Q., Cai N., Li T., Zhang Y., Han Q., Peng Y., Xu H., Lu J., Bao W. (2018). Elucidation of the Molecular-Mechanisms and In Vivo Evaluation of the Anti-inflammatory Effect of Alginate-Derived Seleno-polymannuronate. J. Agric. Food Chem..

[38] Bi D., Lai Q., Han Q., Cai N., He H., Fang W., Yi J., Li X., Xu H., Li X. (2018). Seleno-polymannuronate attenuates neuroin-flammation by suppressing microglial and astrocytic activation. J. Funct. Foods.

[39] Bi D., Lai Q., Li X., Cai N., Li T., Fang W., Han Q., Yu B., Li L., Liu Q. (2019). Neuroimmunoregulatory potential of seleno-polymannuronate derived from alginate in lipopolysaccharide-stimulated BV2 microglia. Food Hydrocoll..

[40] Bi D., Li X., Li T., Li X., Lin Z., Yao L., Li H., Xu H., Hu Z., Zhang Z. (2020). Characterization and Neuroprotection Potential of Seleno-Polymannuronate. Front. Pharmacol..

[41] Zbilenler C., Altundağ E.M., Gazi M. (2020). Synthesis of quercetin-encapsulated alginate beads with their antioxidant and release kinetic studies. J. Macromol. Sci. Part A.

[42] Lević S., Lijaković I.P., Đorđević V., Rac V., Rakić V., Knudsen T.Š., Pavlović V., Bugarski B., Nedović V. (2015). Characterization of sodium alginate/d-limonene emulsions and respective calcium alginate/d-limonene beads produced by electrostatic ex-trusion. Food Hydrocoll..

[43] Mørch Ý.A., Donati I., Strand B.L., Skjåk-Bræk G. (2006). Effect of Ca^2+^, Ba^2+^, and Sr^2+^ on alginate microbeads. Biomacromolecules.

[44] Draget K.I., Bræk G.S., Smidsrød O. (1994). Alginic acid gels: The effect of alginate chemical composition and molecular weight. Carbohydr. Polym..

[45] Liu X.D., Yu W.Y., Zhang Y., Xue W.M., Xiong Y., Ma X.J., Chen Y., Yuan Q. (2002). Characterization of structure and diffusion behaviour of Ca-alginate beads prepared with external or internal calcium sources. J. Microencapsul..

[46] Draget K.I. (2009). Alginates. Handbook of Hydrocolloids.

[47] Chen H., Lu Y., Yuan F., Gao Y., Mao L. (2021). Effect of interfacial compositions on the physical properties of alginate-based emulsion gels and chemical stability of co-encapsulated bioactives. Food Hydrocoll..

[48] Li P., Guo C., Li X., Yuan K., Yang X., Guo Y., Yang X. (2021). Preparation and structural characteristics of composite algi-nate/casein emulsion gels: A microscopy and rheology study. Food Hydrocoll..

[49] Puguan J.M.C., Yu X., Kim H. (2014). Characterization of structure, physico-chemical properties and diffusion behavior of Ca-Alginate gel beads prepared by different gelation methods. J. Colloid Interface Sci..

[50] Jeong C., Kim S., Lee C., Cho S., Kim S.-B. (2020). Changes in the Physical Properties of Calcium Alginate Gel Beads under a Wide Range of Gelation Temperature Conditions. Foods.

[51] Kim S., Jeong C., Cho S., Kim S.-B. (2019). Effects of thermal treatment on the physical properties of edible calcium alginate gel beads: Response surface methodological approach. Foods.

[52] Wang X., Feng Y., Feng T., Wang X., Xia S., Zhang X. (2021). Modulation effect of glycerol on plasticization and water distribution of vacuum-dried calcium alginate gel beads encapsulating peppermint oil/β-cyclodextrin complex. Food Biosci..

[53] Puguan J.M.C., Yu X., Kim H. (2015). Diffusion characteristics of different molecular weight solutes in Ca–alginate gel beads. Colloids Surfaces A Physicochem. Eng. Asp..

[54] Lopez-Sanchez P., Fredriksson N., Larsson A., Altskär A., Ström A. (2018). High sugar content impacts microstructure, mechanics and release of calcium-alginate gels. Food Hydrocoll..

[55] Stojanovic R., Belscak-Cvitanovic A., Manojlovic V., Komes D., Nedovic V., Bugarski B. (2012). Encapsulation of thyme (*Thymus serpyllum* L.) aqueous extract in calcium alginate beads. J. Sci. Food Agric..

[56] Najafi-Soulari S., Shekarchizadeh H., Kadivar M. (2016). Encapsulation optimization of lemon balm antioxidants in calcium al-ginate hydrogels. J. Biomater. Sci. Polym. Ed..

[57] Kim E.S., Lee J.-S., Lee H.G. (2016). Calcium-alginate microparticles for sustained release of catechin prepared via an emulsion gelation technique. Food Sci. Biotechnol..

[58] Sohail A., Turner M.S., Coombes A.G., Bostrom T., Bhandari B. (2011). Survivability of probiotics encapsulated in alginate gel microbeads using a novel impinging aerosols method. Int. J. Food Microbiol..

[59] Petzold G., Gianelli M.P., Bugueño G., Celan R., Pavez C., Orellana P. (2014). Encapsulation of liquid smoke flavoring in ca-alginate and ca-alginate-chitosan beads. J. Food Sci. Technol..

[60] Heidebach T., Först P., Kulozik U. (2012). Microencapsulation of Probiotic Cells for Food Applications. Crit. Rev. Food Sci. Nutr..

[61] Donati I., Paoletti S. (2009). Material properties of alginates. Alginates: Biology and Applications.

[62] Draget K.I., Strand B., Hartmann M., Valla S., Smidsrød O., Skjåk-Bræk G. (2000). Ionic and acid gel formation of epimerised alginates; the effect of AlgE4. Int. J. Biol. Macromol..

[63] Draget K.I., Skjåk-Bræk G., Stokke B.T. (2006). Similarities and differences between alginic acid gels and ionically crosslinked alginate gels. Food Hydrocoll..

[64] Atkins E., Mackie W., Parker K., Smolko E. (1971). Crystalline structures of poly-D-mannuronic and poly-L-guluronic acids. J. Polym. Sci. Part B Polym. Lett..

[65] Malmud L.S., Charkes N.D., Littlefield J., Reilley J., Stern H., Rosenberg R., Fisher R.S. (1979). The mode of action alginic acid compound in the reduction of gastroesophageal reflux. J. Nucl. Med..

[66] Foocharoen C., Chunlertrith K., Mairiang P., Mahakkanukrauh A., Suwannaroj S., Namvijit S., Wantha O., Nanagara R. (2017). Effectiveness of add-on therapy with domperidone vs alginic acid in proton pump inhibitor partial response gas-tro-oesophageal reflux disease in systemic sclerosis: Randomized placebo-controlled trial. Rheumatology.

[67] Lin D., Kelly A.L., Maidannyk V., Miao S. (2020). Effect of concentrations of alginate, soy protein isolate and sunflower oil on water loss, shrinkage, elastic and structural properties of alginate-based emulsion gel beads during gelation. Food Hydrocoll..

[68] Lin D., Kelly A.L., Miao S. (2021). The role of mixing sequence in structuring O/W emulsions and emulsion gels produced by electrostatic protein-polysaccharide interactions between soy protein isolate-coated droplets and alginate molecules. Food Hydrocoll..

[69] Leon A.M., Medina W.T., Park D.J., Aguilera J.M. (2018). Properties of microparticles from a whey protein isolate/alginate emulsion gel. Food Sci. Technol. Int..

[70] Lin D., Kelly A.L., Maidannyk V., Miao S. (2020). Effect of structuring emulsion gels by whey or soy protein isolate on the structure, mechanical properties, and in-vitro digestion of alginate-based emulsion gel beads. Food Hydrocoll..

[71] Deng Z., Li J., Song R., Zhou B., Li B., Liang H. (2021). Carboxymethylpachymaran/alginate gel entrapping of natural pollen capsules for the encapsulation, protection and delivery of probiotics with enhanced viability. Food Hydrocoll..

[72] Yan J., Liang X., Ma C., McClements D.J., Liu X., Liu F. (2021). Design and characterization of double-cross-linked emulsion gels using mixed biopolymers: Zein and sodium alginate. Food Hydrocoll..

[73] Li A., Gong T., Hou Y., Yang X., Guo Y. (2020). Alginate-stabilized thixotropic emulsion gels and their applications in fabrication of low-fat mayonnaise alternatives. Int. J. Biol. Macromol..

[74] Yang X., Gong T., Lu Y.-H., Li A., Sun L., Guo Y. (2020). Compatibility of sodium alginate and konjac glucomannan and their applications in fabricating low-fat mayonnaise-like emulsion gels. Carbohydr. Polym..

[75] Yang X., Li A., Yu W., Li X., Sun L., Xue J., Guo Y. (2020). Structuring oil-in-water emulsion by forming egg yolk/alginate complexes: Their potential application in fabricating low-fat mayonnaise-like emulsion gels and redispersible solid emul-sions. Int. J. Biol. Macromol..

[76] Li Y., Lu J., Tian X., Xu Z., Huang L., Xiao H., Ren X., Kong Q. (2021). Alginate with citrus pectin and pterostilbene as healthy food packaging with antioxidant property. Int. J. Biol. Macromol..

[77] Umaraw P., Verma A.K. (2017). Comprehensive review on application of edible film on meat and meat products: An eco-friendly approach. Crit. Rev. Food Sci. Nutr..

[78] Maizura M., Fazilah A., Norziah M., Karim A. (2007). Antibacterial Activity and Mechanical Properties of Partially Hydrolyzed Sago Starch? Alginate Edible Film Containing Lemongrass Oil. J. Food Sci..

[79] Ismillayli N., Hadi S., Dharmayani N.K.T., Sanjaya R.K., Hermanto D. (2020). Characterization of Alginate-Chitosan Membrane as Potential Edible Film.

[80] Reyes-Avalos M.C., Femenia A., Minjares-Fuentes R., Contreras-Esquivel J.C., Aguilar-González C.N., Esparza-Rivera J.R., Meza-Velázquez J.A. (2016). Improvement of the Quality and the Shelf Life of Figs (*Ficus carica*) Using an Alginate-Chitosan Edible Film. Food Bioprocess Technol..

[81] Reyes-Avalos M., Minjares-Fuentes R., Femenia A., Contreras-Esquivel J., Quintero-Ramos A., Esparza-Rivera J., Meza-Velázquez J. (2019). Application of an Alginate–Chitosan Edible Film on Figs (*Ficus carica*): Effect on Bioactive Compounds and Antioxidant Capacity. Food Bioprocess Technol..

[82] Kazemi S.M., Rezaei M. (2015). Antimicrobial Effectiveness of Gelatin-Alginate Film Containing Oregano Essential Oil for Fish Preservation. J. Food Saf..

[83] Dou L., Li B., Zhang K., Chu X., Hou H. (2018). Physical properties and antioxidant activity of gelatin-sodium alginate edible films with tea polyphenols. Int. J. Biol. Macromol..

[84] Bastos D.D.S., Araújo K.G.D.L., Leão M.H.M.D.R. (2009). Ascorbic acid retaining using a new calcium alginate-Capsul based edible film. J. Microencapsul..

[85] Ruan C., Zhang Y., Wang J., Sun Y., Gao X., Xiong G., Liang J. (2019). Preparation and antioxidant activity of sodium alginate and carboxymethyl cellulose edible films with epigallocatechin gallate. Int. J. Biol. Macromol..

[86] Martínez-Molina E.C., Freile-Pelegrín Y., Ovando-Chacón S.L., Gutiérrez-Miceli F.A., Ruiz-Cabrera M.Á., Grajales-Lagunes A., Luján-Hidalgo M.C., Abud-Archila M. (2021). Development and characterization of alginate-based edible film from *Sargassum fluitans* incorporated with silver nanoparticles obtained by green synthesis. J. Food Meas. Charact..

[87] Lim L.I., Tan H.L., Pui L.P. (2021). Development and characterization of alginate-based edible film incorporated with hawthorn berry (*Crataegus pinnatifida*) extract. J. Food Meas. Charact..

[88] Kuan Y.L., Sivanasvaran S.N., Pui L.P., Yusof Y.A., Senphan T. (2020). Physicochemical Properties of Sodium Alginate Edible Film Incorporated with Mulberry (*Morus australis*) Leaf Extract. Pertanika J. Trop. Agric. Sci..

[89] Zhu D., Guo R., Li W., Song J., Cheng F. (2019). Improved Postharvest Preservation Effects of *Pholiota nameko* Mushroom by Sodium Alginate–Based Edible Composite Coating. Food Bioprocess Technol..

[90] Mahcene Z., Khelil A., Hasni S., Bozkurt F., Goudjil M.B., Tornuk F. (2020). Home-made cheese preservation using sodium alginate based on edible film incorporating essential oils. J. Food Sci. Technol..

[91] Zhang S., Wei F., Han X. (2018). An edible film of sodium alginate/pullulan incorporated with capsaicin. New J. Chem..

[92] Kleinert M., Clemmensen C., Hofmann S.M., Moore M.C., Renner S., Woods S.C., Huypens P., Beckers J., de Angelis M.H., Schürmann A. (2018). Animal models of obesity and diabetes mellitus. Nat. Rev. Endocrinol..

[93] Jensen M.G., Kristensen M., Astrup A. (2012). Effect of alginate supplementation on weight loss in obese subjects completing a 12-wk energy-restricted diet: A randomized controlled trial. Am. J. Clin. Nutr..

[94] Wilcox M.D., Brownlee I.A., Richardson J.C., Dettmar P.W., Pearson J.P. (2014). The modulation of pancreatic lipase activity by alginates. Food Chem..

[95] Houghton D., Wilcox M.D., Chater P.I., Brownlee I.A., Seal C.J., Pearson J.P. (2015). Biological activity of alginate and its effect on pancreatic lipase inhibition as a potential treatment for obesity. Food Hydrocoll..

[96] Wilcox M.D., Chater P.I., Stanforth K.J., Woodcock A.D., Dettmar P.W., Pearson J.P. (2021). The rheological properties of an alginate satiety formulation in a physiologically relevant human model gut system. Ann. Esophagus.

[97] Guo L., Goff H.D., Xu F., Liu F., Ma J., Chen M., Zhong F. (2020). The effect of sodium alginate on nutrient digestion and metabolic responses during both in vitro and in vivo digestion process. Food Hydrocoll..

[98] Odunsi S.T., Vázquez-Roque M.I., Camilleri M., Papathanasopoulos A., Clark M.M., Wodrich L., Lempke M., McKinzie S., Ryks M., Burton D. (2010). Effect of alginate on satiation, appetite, gastric function, and selected gut satiety hormones in over-weight and obesity. Obesity.

[99] Liu J., Wu S., Cheng Y., Liu Q., Su L., Yang Y., Zhang X., Wu M., Choi J.-I., Tong H. (2021). *Sargassum fusiforme* Alginate Relieves Hyperglycemia and Modulates Intestinal Microbiota and Metabolites in Type 2 Diabetic Mice. Nutrients.

[100] Lee K.H., Song Y., Wu W., Yu K., Zhang G. (2020). The gut microbiota, environmental factors, and links to the development of food allergy. Clin. Mol. Allergy.

[101] Kesika P., Suganthy N., Sivamaruthi B.S., Chaiyasut C. (2021). Role of gut-brain axis, gut microbial composition, and probiotic intervention in Alzheimer's disease. Life Sci..

[102] Rastelli M., Knauf C., Cani P.D. (2018). Gut Microbes and Health: A Focus on the Mechanisms Linking Microbes, Obesity, and Related Disorders. Obesity.

[103] Singer-Englar T., Barlow G., Mathur R. (2019). Obesity, diabetes, and the gut microbiome: An updated review. Expert Rev. Gastroenterol. Hepatol..

[104] Yao L., Yang P., Lin Y., Bi D., Yu B., Lin Z., Wu Y., Xu H., Hu Z., Xu X. (2021). The regulatory effect of alginate on ovalbu-min-induced gut microbiota disorders. J. Funct. Foods.

[105] Huang J., Huang J., Li Y., Wang Y., Wang F., Qiu X., Liu X., Li H. (2021). Sodium Alginate Modulates Immunity, Intestinal Mucosal Barrier Function, and Gut Microbiota in Cyclophosphamide-Induced Immunosuppressed BALB/c Mice. J. Agric. Food Chem..

[106] Ejima R., Akiyama M., Sato H., Tomioka S., Yakabe K., Kimizuka T., Seki N., Fujimura Y., Hirayama A., Fukuda S. (2021). Seaweed Dietary Fiber Sodium Alginate Suppresses the Migration of Colonic Inflammatory Monocytes and Diet-Induced Metabolic Syndrome via the Gut Microbiota. Nutrients.

[107] Ai C., Jiang P., Liu Y., Duan M., Sun X., Luo T., Jiang G., Song S. (2019). The specific use of alginate from Laminaria japonica by Bacteroides species determined its modulation of the Bacteroides community. Food Funct..

[108] Al-Najjar M.A.A., Athamneh T., AbuTayeh R., Basheti I., Leopold C., Gurikov P., Smirnova I. (2021). Evaluation of the orally administered calcium alginate aerogel on the changes of gut microbiota and hepatic and renal function of Wistar rats. PLoS ONE.

[109] You L., Gong Y., Li L., Hu X., Brennan C., Kulikouskaya V. (2020). Beneficial effects of three brown seaweed polysaccharides on gut microbiota and their structural characteristics: An overview. Int. J. Food Sci. Technol..

[110] Campos-Perez W., Martinez-Lopez E. (2021). Effects of short chain fatty acids on metabolic and inflammatory processes in human health. Biochim. Biophys. Acta (BBA) Mol. Cell Biol. Lipids.

[111] Gordon S. (2002). Pattern recognition receptors: Doubling up for the innate immune response. Cell.

[112] Kurachi M., Nakashima T., Yamaguchi K., Oda T., Miyajima C., Iwamoto Y., Muramatsu T. (2005). Comparison of the activities of various alginates to induce TNF-α secretion in RAW264.7 cells. J. Infect. Chemother..

[113] Yang D., Jones K.S. (2009). Effect of alginate on innate immune activation of macrophages. J. Biomed. Mater. Res. Part A.

[114] Bi D., Zhou R., Cai N., Lai Q., Han Q., Peng Y., Jiang Z., Tang Z., Lu J., Bao W. (2017). Alginate enhances Toll-like receptor 4-mediated phagocytosis by murine RAW264.7 macrophages. Int. J. Biol. Macromol..

[115] Jeong H., Lee S., Moon P., Na H., Park R., Um J., Kim H., Hong S. (2006). Alginic acid has anti-anaphylactic effects and inhibits inflammatory cytokine expression via suppression of nuclear factor-κB activation. Clin. Exp. Allergy.

[116] Yu B., Bi D., Yao L., Li T., Gu L., Xu H., Li X., Li H., Hu Z.-L., Xu X. (2020). The inhibitory activity of alginate against allergic reactions in an ovalbumin-induced mouse model. Food Funct..

[117] Fujihara M., Iizima N., Yamamoto I., Nagumo T. (1984). Purification and chemical and physical characterisation of an antitumour polysaccharide from the brown seaweed Sargassum fulvellum. Carbohydr. Res..

[118] Fujihara M., Nagumo T. (1992). The effect of the content of d-mannuronic acid and l-guluronic acid blocks in alginates on antitumor activity. Carbohydr. Res..

[119] Fujihara M., Nagumo T. (1993). An influence of the structure of alginate on the chemotactic activity of macrophages and the anti-tumor activity. Carbohyd. Res..

